# Supplementary material for: A Novel Mechanism for Bone Loss: Platelet Count Negatively Correlates with Bone Mineral Density via Megakaryocyte-Derived RANKL
Source: Int J Mol Sci. 2023 Jul 29;24(15):12150. doi: 10.3390/ijms241512150 (PMC10418703; doi:10.3390/ijms241512150)
Supplement: Supplementary file 1 [file ijms-24-12150-s001.zip › ijms-2514624-supplementary.pdf]

Table S1. The PCR primers for M-CSF, OPG, RANKL, and GAPDH.

Genes	Primers	
	Forward	Reverse
RANKL	5’-GCC TTT CAA GGA GCT GTG CAA AA-3’	5’-GAG CAA AAG GCT GAG CTT CAA GC-3’
OPG	5’-GGT CTC CTG CTA ACT CAG AAA GG-3’	5’-CAG CAA ACC TGA AGA ATG CCT CC-3’
M-CSF	5’-TGA GAC ACC TCT CCA GTT GCT G-3’	5’-GCA ATC AGG CTT GGT CAC CAC A-3’
GAPDH	5’-GTC TCC TCT GAC TTC AAC AGC G-3’	5’-ACC ACC CTG TTG CTG TAG CCA A-3’
